# Central Venous Catheter–associated *Nocardia* Bacteremia in Cancer Patients

**DOI:** 10.3201/eid1709.101810

**Published:** 2011-09

**Authors:** Fadi Al Akhrass, Ray Hachem, Jamal A. Mohamed, Jeffrey Tarrand, Dimitrios P. Kontoyiannis, Jyotsna Chandra, Mahmoud Ghannoum, Souha Haydoura, Ann Marie Chaftari, Issam Raad

**Affiliations:** Author affiliations: The University of Texas MD Anderson Cancer Center, Houston, Texas, USA (F. Al Akhrass, R. Hachem, J.A. Mohamed, J. Tarrand, D.P. Kontoyiannis, A.M. Chaftari, I. Raad);; Case Western Reserve University, Cleveland, Ohio, USA (J. Chandra, M. Ghannoum);; University Hospitals Case Medical Center, Cleveland (J. Chandra, M. Ghannoum);; Kansas University School of Medicine, Wichita, Kansas, USA (S. Haydoura)

**Keywords:** Nocardia, bacteremia, catheter related, disseminated, immunocompromised, biofilm, cancer, central venous catheter, antimicrobial, bacteria, research

## Abstract

Central venous catheters, often needed by cancer patients, can be the source of *Nocardia* bacteremia. We evaluated the clinical characteristics and outcomes of 17 cancer patients with *Nocardia* bacteremia. For 10 patients, the bacteremia was associated with the catheter; for the other 7, it was a disseminated infection. *N. nova* complex was the leading cause of bacteremia. *Nocardia* promoted heavy biofilm formation on the surface of central venous catheter segments tested in an in vitro biofilm model. Trimethoprim- and minocycline-based lock solutions had potent in vitro activity against biofilm growth. Patients with *Nocardia* central venous catheter–associated bloodstream infections responded well to catheter removal and antimicrobial drug therapy, whereas those with disseminated bacteremia had poor prognoses.

## Abstract 2

TOC Summary: Heavy biofilm growth can be reduced through use of antimicrobial lock solutions.

Nocardiae are partially acid-fast, aerobic, gram-positive, branching filamentous bacteria that are found ubiquitously in soil, fresh water, and marine water (*1*,*2*). *Nocardia* spp. cause serious pulmonary infections (with occasional brain abscesses) in immunocompromised patients, primarily those with cell-mediated immunity abnormalities (*2*,*3*). Nocardiosis most commonly occurs after the organism has been introduced into the respiratory tract, but it may be acquired through direct inoculation into the skin (*4*). However, *Nocardia* bacteremia is rarely reported, even for severely immunocompromised patients with underlying malignancies (*5*,*6*).

Our first objective was to identify the clinical characteristics of *Nocardia* bacteremia and compare the clinical profiles and outcomes for patients with *Nocardia* bacteremia associated with central venous catheters (CVCs), also called central line–associated bloodstream infections (CLABSIs), with those of patients with disseminated *Nocardia* bacteremia. Our second objective was to determine whether *Nocardia* bacteria could form a biofilm on CVCs in a laboratory model and whether biofilm growth could be prevented with the use of antimicrobial lock solutions.

## Patients and Methods

### Clinical Characteristics

By searching the microbiology laboratory database at The University of Texas MD Anderson Cancer Center (Houston, TX, USA) from January 1998 through March 2010, we retrospectively identified 134 episodes of nocardiosis of any sources. *Nocardia* bacteremia was reported for 17 patients; 5 of these cases have been reported by Torres et al. (*7*). In addition, 2 of these 5 patients had catheter-related bloodstream infections (CRBSIs), reported by Kontoyiannis et al. (*8*). Pertinent data from patients’ medical records were abstracted, including demographic characteristics, underlying malignancies, hematopoietic stem cell transplantation, graft-versus-host disease, clinical presentation, laboratory test and imaging study results, concomitant infections, antimicrobial therapy types and durations, hospital and intensive care unit stays, 72-hour and 7-day responses, and patient outcomes at 3-month follow-up.

### Definitions

Neutropenia was defined as an absolute neutrophil count <500 cells/mm^3^ and lymphopenia as an absolute lymphocyte count <1,000 cells/mm^3^. Patients with at least 1 set of positive blood cultures for *Nocardia* bacteria were considered to have *Nocardia* bacteremia. CLABSIs were diagnosed according to Centers for Disease Control and Prevention (Atlanta, GA, USA) guidelines: a recognized pathogen cultured from >1 blood cultures (not including organisms considered common skin contaminants) and no apparent source of the bloodstream infection except the CVC whereby the CVC has been indwelling for >48 hours (*9*). Furthermore, CLABSIs were considered definite CRBSIs if at least 1 of the Infectious Disease Society of America definition criteria were also fulfilled: semiquantitative (>15 CFUs/catheter segment) or quantitative (>10^3^ CFUs/catheter segment) catheter culture in which the same organism is isolated from the catheter segment and peripheral blood, or differential quantitative blood culture with simultaneous quantitative blood cultures from the CVC and peripheral blood with a ratio >3:1 (*10*). Disseminated (or secondary) *Nocardia* bacteremia was defined as the recovery of *Nocardia* bacteria from blood cultures and a non–catheter-related site (e.g., expectorated sputum, endotracheal aspiration, bronchoalveolar lavage, pleural effusion, lung tissue, or skin or brain biopsy samples) in the setting of clinical and radiographic evidence of organ involvement (pneumonia and skin lesions).

*Nocardia* spp. were identified on the basis of the appearance of colonies on routine media; species were identified by using a battery of biochemical tests and, after 2001, by 16S rDNA sequencing (*11*,*12*). Blood cultures and catheter tip samples were held for 7 days to ensure that no cases of *Nocardia* bacteremia were missed.

A broth microdilution MIC method had been used to perform susceptibility testing of *Nocardia* spp. according Clinical and Laboratory Standards Institute guidelines (*13*). Antimicrobial drug response had been defined as resolution or improvement of clinical manifestations and radiographic changes and negative microbiological findings.

### Biofilm Formation

We used a modified Kuhn model of biofilm catheter colonization (*14*) to test *N. nova* complex and *N. puris* strains (that caused CLABSI in this study) for biofilm formation. Sterile polyurethane and silicone CVC segments were placed in 24-well tissue culture plates containing human donor plasma and incubated with shaking for 24 h at 37°C. The plasma was then replaced with 1 mL of 5.5 × 10^5^ cells/mL inoculum of *Nocardia* strains. The *Nocardia* inoculum was grown in tryptic soy broth containing 10% fetal bovine serum and incubated with shaking for 24 h at 37°C. Organisms were tested in triplicate. The inoculated broth was removed, and CVC segments were washed with 1 mL of 0.9% sterile saline by shaking at 100 rpm for 30 min at 37°C. The CVC segments were transferred into a tube containing 5 mL of sterile 0.9% saline and sonicated for 15 min to disrupt any biofilm. The resulting solution was then cultured and quantified by making serial dilutions in 0.9% sterile saline and spreading them on trypticase soy agar plates with 5% sheep blood. All plates were inverted and incubated for 48 h at 37°C. The experiment was repeated 2 times.

### Antimicrobial Lock Solutions

To determine whether antimicrobial lock solutions prevented the biofilm growth of *Nocardia* organisms, we used the silicone disk biofilm colonization model, as described (*14*). After washing the silicone disks with 0.9% sterile saline by shaking them for 30 min at 37°C, we transferred the disks into new 24-well tissue culture plates containing Mueller-Hinton broth (control) or the drug solution to be tested. Drug solutions included 10 mg/mL trimethoprim/sulfamethoxazole and 100 U heparin; a triple combination of 10 mg/mL trimethoprim, 30 mg/mL EDTA, and 25% ethanol; and 3 mg/mL minocycline, 30 mg EDTA, and 25% ethanol. Triple combinations of minocycline, EDTA, and 25% ethanol lock solutions were used because previous data showed that such combinations effectively eradicate bacterial organisms embedded in biofilm (*15*). After 2 h of incubation at 37°C, the disks were placed in 5 mL of 0.9% saline and sonicated for 15 min. Finally, they were vortexed for 5 seconds, and 100 µL of liquid from each disk was serially diluted and spread on trypticase soy agar plates with 5% sheep blood for quantitative culture. Plates were then inverted and incubated for 48–72 h at 37°C, and colony growth was quantified. The experiment was repeated 2 times.

### Electronic Microscopy and Confocal Scanning

To verify the quantitative results, we used scanning electron microscopy to examine biofilm formation of *N. nova* complex on silicone CVC surfaces. We used silicone CVCs tested in the in vitro colonization model outlined above**.** Catheters were fixed with 2% glutaraldehyde, followed by osmium tetraoxide, tannic acid, and uranyl acetate as described (*16*). A series of ethanol dehydration steps followed, and the prepared samples were sputter coated with Au-Pd (60:40) and viewed with a Philips model XL3C scanning electron microscope (Philips Research, Eindhoven, the Netherlands). Confocal scanning laser microscopy was performed with a Leica TCSNT confocal microscope (Leica, Heidelberg, Germany). Objectives used for confocal laser microscope imaging were 100×1.4 N.A. Oil Plan Apo and 63×0.7 N.A. Plan Fluotar.

### Statistical Analyses

To compare the characteristics of *Nocardia* CLABSI and disseminated *Nocardia* bacteremia patients, we used SAS version 9.1 (SAS Institute, Inc., Cary, NC, USA). We determined differences between categorical variable frequencies by using the χ^2^ or Fisher exact tests and compared continuous variables by using the Wilcoxon rank-sum test. All tests were 2-sided and had a maximum significance level of 0.05.

## Results

The demographic characteristics and outcomes of the 17 patients in the study are shown in Table 1. All patients had had CVCs inserted before examination and collection of blood for culture. Ten (59%) patients had CLABSIs (Table 2); the remaining 7 (41%) had disseminated (secondary) *Nocardia* bacteremia for which the respiratory tract (pneumonia) was the primary source. Concomitant infections, most commonly with cytomegalovirus, were noted for 10 (59%) patients. Hematologic malignancies were more common in patients with disseminated *Nocardia* bacteremia than with *Nocardia* CLABSIs (100% vs. 50%; p = 0.044). *N. nova* complex was the most commonly identified causative species for nocardemia (6 patients). Other causative species were *N. asteroides* complex (5 patients), *N. veterana* (2), *N. brasiliensis* (1), and *N. puris* (1). We found no significant differences in species distribution among patients with CLABSIs and disseminated bacteremia. Furthermore, by looking at the temporal distribution of cases of *Nocardia* bacteremia and CLABSI nocardemia, we noticed no cluster that would suggest we were dealing with an outbreak.

**Table 1:** Characteristics and outcomes of 17 cancer patients with *Nocardia* bacteremia, The University of Texas MD Anderson Cancer Center, January 1998–March 2010*

Characteristic	Disseminated bacteremia (n = 7), no. (%) patients	CLABSI (n = 10), no. (%) patients	p value
Male sex	3 (43)	4 (40)	>0.99
Cancer type			0.044
Hematologic	7 (100)	5 (50)	
Solid tumor	0	5 (50)	
Concomitant infection	7 (100)	5 (50)	0.044
Cytomegalovirus	4 (57)	1 (10)	0.1
Invasive fungus	3 (42)	1 (10)	0.25
*Nocardia* type†			0.74
*N. nova* complex	3 (43)	3 (30)	
*N. asteroides* complex	1 (14 )	4 (40)	
* N. veterana*	1 (17)	1 (11)	
* N. brasiliensis*	1 (17)	0	
* N. puris*	0	1 (11)	
Antimicrobial drug treatment outcome			
Response to therapy	5 (71)	9 (90)	0.54
Response within 72 h	0	9 (90)	<0.001
Response within 7 d	2 (29)	9 (90)	0.035
Death within 3 mo	3 (43)	1 (10)	0.25
*Nocardia* bacteremia breakthrough despite trimethoprim/sulfamethoxazole prophylaxis	1	2	>0.99

*CLABSI, central line–associated bloodstream infection. Patient median age (range) for those with disseminated infection 56 y (32–73 y), for those with CLABSI 44 y (11–77 y); p = 0.31. Median hospital stay (range) for those with disseminated infection 24 d (8–53 d), for those with CLABSI 5 d (2–18 d); p = 0.01. Median antimicrobial drug treatment duration (range) for those with disseminated infection 50 d (21–105 d), for those with CLABSI 82 d (14–120 d); p = 0.56. †Species were not determined for 2 isolates.

**Table 2:** Diagnostic and microbiologic profile for 10 cases of *Nocardia* CLABSI, The University of Texas MD Anderson Cancer Center, January 1998–March 2010*

CLABSI case no.	Diagnostic criteria	CRBSI
1	Differential quantitative blood culture (CVC >1,000 CFU/mL; peri = 3 CFU/mL) and quantitative catheter tip culture (10^3^ CFU/tip)	Definite
2	Positive peripheral blood culture with positive semi-quantitative catheter tip culture (>15 CFU/tip)	Definite
3	Differential quantitative blood culture (CVC >1,000 CFUs/mL; peri = 1 CFU/mL)	Definite
4	CVC blood culture positive with positive semiquantitative catheter tip culture (>15 CFU/tip)	Definite
5	Differential quantitative blood culture (CVC >1,000 CFU/mL; peri = 1 CFU/mL)	Definite
6	Positive peripheral and CVC blood cultures with positive quantitative catheter tip culture (4,000 CFU/tip)	Definite
7	Differential quantitative blood culture (CVC >1,000 CFU/mL; peri = 20 CFUs/mL) and positive quantitative catheter tip culture (4,000 CFU/tip)	Definite
8	Positive peripheral and CVC blood culture but negative catheter tip culture	Probable
9	Positive peripheral and CVC blood culture but negative catheter tip culture	Probable
10	Positive CVC and peripheral blood culture but negative catheter tip culture	Probable

*CLABSI, central line–associated bloodstream infection; CRBSI, catheter-related bloodstream infection, defined according to Infectious Disease Society of America guidelines (*10*); CVC, central venous catheter; peri, peripheral. CVC values indicate blood for culture collected from CVC; peri values indicate blood for culture collected from peripheral vessel.

Patients with CLABSIs had shorter hospital stays than did those with disseminated bacteremia (median 5 days vs. 24 days, respectively; p = 0.01) and were more likely to experience a response to therapy within 72 hours (90% vs. 0%; p<0.001) or 7 days (90% vs. 29%; p = 0.035). Mortality rate at 3-month follow-up was also lower for patients with CLABSI (10% vs. 43%; p = 0.25).

All *Nocardia* isolates were susceptible to trimethoprim/sulfamethoxazole, amikacin, and linezolid. Susceptibility rates to ceftriaxone, clarithromycin, imipenem, minocycline, tobramycin, and amoxicillin and clavulanate were 91%, 87%, 85%, 55%, 42%, and 22%, respectively. All *N. nova* isolates were susceptible to clarithromycin. No resistance to minocycline was found, but 45% of isolates had intermediate susceptibility MICs. *N. nova* complex isolates showed resistance to amoxicillin and clavulanate and to gentamicin in 80% and 60% of cases, respectively, whereas 91% of all *Nocardia* species were resistant to ciprofloxacin.

All patients underwent CVC removal. The most commonly used drug was trimethoprim/sulfamethoxazole (14 patients), used alone or in combination with beta-lactams (8 patients with carbapenems and 2 with ceftriaxone), minocycline (5 patients), or amikacin (4 patients). A positive response occurred in 14 patients, and breakthrough *Nocardia* bacteremia occurred in 3 patients receiving trimethoprim/sulfamethoxazole prophylaxis (2 with CLABSIs and 1 with disseminated bacteremia).

**Figure 1:**
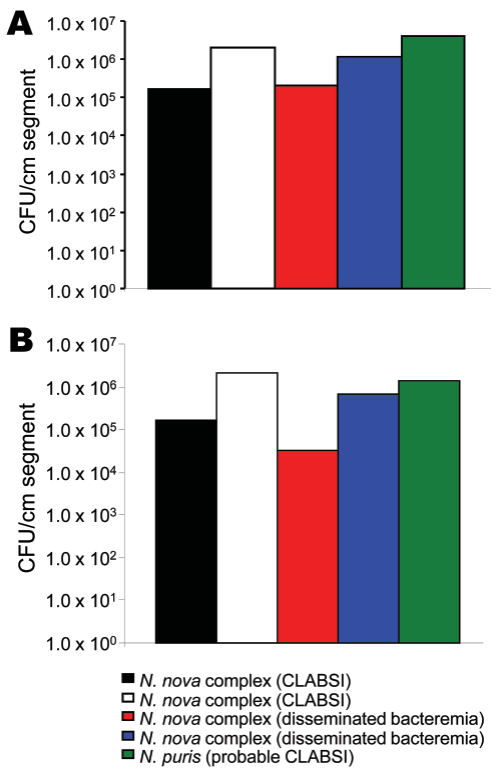
*Nocardia nova* and *N.* *puris* quantitative biofilm formation, as assessed by biofilm colonization model. *Nocardia* spp. isolates adhered to polyurethane (A) and silicone (B) central venous catheter segments with extensive biofilms. CLABSI, central line–associated bloodstream infection.

**Figure 2:**
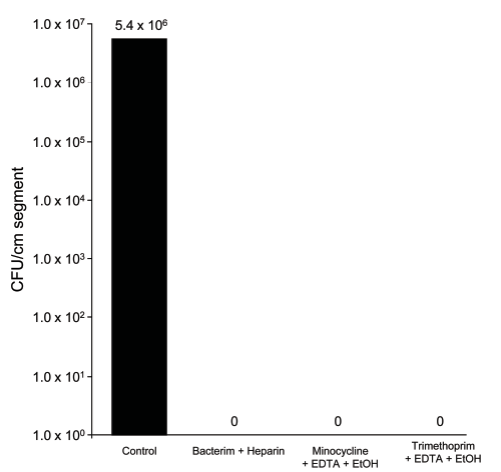
Antibiofilm agents inhibition of biomass of *Nocardia nova* complex biofilms. *N. nova* complex biofilms were grown for 24 h on silicone disks, placed in 24-well tissue culture plates, and exposed to trimethoprim/sulfamethoxazole (Bacterim) and heparin; trimethoprim, EDTA, and ethanol (EtOH); minocycline, EDTA, and ethanol; or Mueller-Hinton broth medium (control) for 2 h. Minocycline and trimethoprim-based lock solutions completely inhibited the *N. nova* complex biofilm biomass compared with controls (p = 0.003).

*Nocardia* isolates adhered extensively to polyurethane and silicone CVCs and formed extensive biofilms (Figure 1). Quantitative biofilm cultures isolated ranged from 4.0 × 10^5^ to 1.1 × 10^7^ CFUs of tested *Nocardia* cells from biofilm matrices that adhered to polyurethane and silicone CVC surfaces. Furthermore, trimethoprim/sulfamethoxazole with heparin, minocycline, and EDTA in 25% ethanol, and trimethoprim and EDTA in 25% ethanol significantly decreased the biofilm biomasses on treated vs. control plates after a 2-hour exposure (p = 0.003), resulting in complete eradication of *Nocardia* spp. in biofilm on silicone disks (Figure 2).

**Figure 3:**
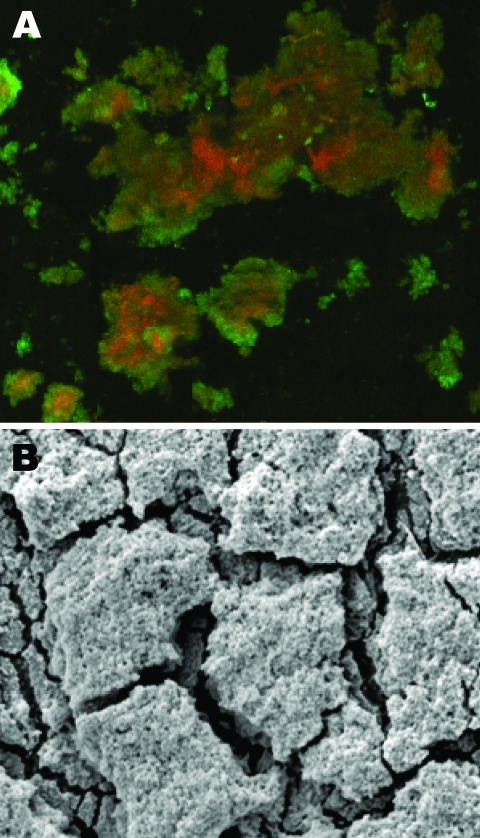
A) Confocal scanning laser microscopy image of central venous catheter tip in a patient with *Nocardia nova* complex central line–associated bloodstream infection. Bright green objects are viable biofilm bacteria, and orange-red objects are dead bacteria. Original magnification ×25. B) Scanning electron microscopy image of central venous catheter tip reveals biofilm surface structure. Original magnification ×5,000.

**Figure 4:**
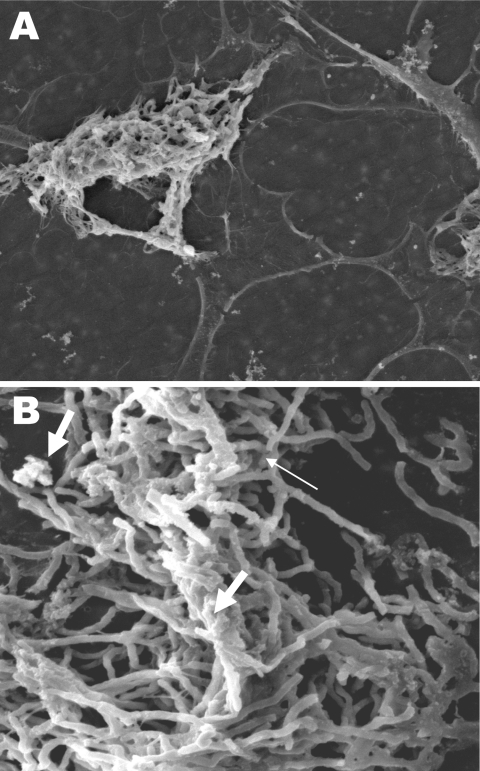
Scanning electron microscopy of *Nocardia* spp. biofilm on silicone central venous catheters. A) *N. nova* complex (disseminated *Nocardia* bacteremia) on the surface, showing heavy biofilm matrix covering filamentous cells. B) *N. nova* complex (definite central line–associated bloodstream infection) showing network of filamentous (thin arrow), partially covered with opaque biofilm matrix (thick arrows). Original magnifications ×2,500.

Electron and confocal scanning laser microscopic studies of the CVC tip in a patient with an *N. nova* CLABSI showed *Nocardia* spp. adhering to the surface and colonies of multilayered clusters embedded in biofilm matrix (Figure 3). Scanning electron microscopy studies of *N. nova* complex (disseminated *Nocardia* bacteremia) showed a heavy biofilm matrix covering filamentous clusters (Figure 4, panel A). *N. nova* complex (definite CLABSI) formed branching networks of filamentous bacteria encompassed in an intense heavy biofilm matrix (Figure 4, panel B).

## Discussion

We have documented that *Nocardia* spp. that cause clinical CLABSI also form heavy biofilm on the surfaces of polyurethane and silicone CVCs; that trimethoprim- and minocycline-based lock solutions eradicate adherent catheter-related *Nocardia* spp. in the biofilm matrix (thereby demonstrating Koch’s postulate as it relates to *Nocardia* CLABSI); and that *Nocardia* bacteremia in cancer patients can occur as CLABSI in 59% of cases. *Nocardia* CLABSI responds well to antimicrobial agents and CVC removal and is associated with a short hospital stay, whereas disseminated *Nocardia* bacteremia is associated with a poor prognosis.

Identification of *Nocardia* is more rapid, precise, and accurate with PCR and 16S rDNA sequencing than with standard phenotypic techniques (*17*–*19*). *N. asteroides* complex, which includes *N. farcinica*, *N. nova*, and *N. asteroides* sensu stricto, is the most commonly identified causative species in cancer patients (*7*). In our study, *N. nova* complex was responsible for 35% of *Nocardia* bacteremia episodes. *N. nova* is differentiated from other members of *N. asteroides* complex by DNA homologic characteristics; it is more appropriate to refer to *N. nova* as *N. nova* complex because it comprises 4 distinct species (*12*,*17*,*20*). Furthermore, the incidence of *N. nova* complex bacteremia may have been underestimated in our study because the species was not identified for 5 cases of infection with *N. asteroides* complex. *N. nova* complex strains may have higher tendencies toward CVC adherence, biofilm formation, and hematogenous spread.

*Nocardia* bacteremia is rare, even in severely immunocompromised patients with underlying malignancies (*11*). A 1998 review of the medical literature found only 36 cases of *Nocardia* bacteremia worldwide over 52 years (*5*). All *Nocardia* bacteremia patients in our study had indwelling CVCs before diagnosis and subsequently had catheters removed. Cancer patients depend immensely on vascular access devices, and CVCs may be the source and focus of *Nocardia* bacteremia, given the ubiquitous presence of these organisms in the environment, their possible acquisition through the skin, and their ability to adhere to CVCs through biofilm formation on the surface of catheters as shown in our study (Figure 1) (*7*,*21*,*22*).

*Nocardia* CLABSI cases, in contrast to disseminated *Nocardia* bacteremia cases, were associated with a favorable outcome. Catheter-related *Nocardia* bacteremia might be less likely to invade remote anatomical structures and form deep-seated infections. Disseminated *Nocardia* bacteremia, on the other hand, was associated with higher rates of admission to intensive care units, more frequent occurrence with hematologic malignancy, longer hospital stays, and lower antimicrobial therapy response rates. Factors that could have contributed to the poor outcome include involvement of the lungs and other organs; severity of the underlying disease (hematologic malignancy); and a higher rate of concomitant infections, most commonly with cytomegalovirus and invasive fungi (Table 1).

Although trimethoprim/sulfamethoxazole was uniformly active against *Nocardia* spp. and the most commonly used drug for *Nocardia* bacteremia, breakthrough *Nocardia* bacteremia occurred in 3 patients despite receipt of trimethoprim/sulfamethoxazole prophylaxis. Given that *Nocardia* spp. were shown in our model to form an antimicrobial drug–resistant multilayered biofilm matrix, in which they embed themselves, it is not surprising that 2 of the 3 cases of breakthrough *Nocardia* bacteremia that occurred during trimethoprim/sulfamethoxazole prophylaxis were CLABSIs. CVC biofilm colonization through formation of an antimicrobial drug–resistant matrix is the main factor in the pathogenesis of CRBSIs. The biofilm enables *Nocardia* spp. to protect themselves from the relatively low serum concentrations of antimicrobial drugs given orally and, hence, create a foothold from which they can invade the bloodstream through the surface of the CVC intravascular segment. Prophylactic antimicrobial drugs given orally are ineffective at breaking down the matrix and eradicating the bacteria, which poses a therapeutic and prophylactic challenge for cancer patients with a CVC. Furthermore, trimethoprim/sulfamethoxazole has been ineffective when used alone, especially against disseminated forms of nocardiosis (*23*).

Intraluminal antimicrobial lock therapy has been proposed for the prevention and treatment of CLABSIs (*24*–*27*). For antimicrobial lock therapy, the catheter lumen is filled with 2–4 mL of antimicrobial solution at a concentration 100- to 1,000-fold higher than the MIC of the drug or its usual target systemic concentration; to eradicate the organisms embedded in the intraluminal biofilm, the solution is then allowed to dwell (lock) while the catheter is not in use (*27*). We found antimicrobial catheter lock solutions with active agents such as trimethoprim/sulfamethoxazole or minocycline to be effective against *Nocardia* biofilm. Use of these solutions might be a valid way to prevent *Nocardia* CLABSIs and salvage the CVC, particularly in cancer patients with CLABSI, for whom removal of the CVC might not be possible because of severe thrombocytopenia or lack of other vascular access.

Although no complete correlation exists between in vitro susceptibility and clinical outcome, antimicrobial drug susceptibility tests should be performed for *Nocardia* isolates in immunocompromised patients to guide therapy. Drug susceptibility of *Nocardia* spp. varies among species (*22*).

In agreement with previous results, our findings also confirmed in vitro activity of trimethoprim/sulfamethoxazole, amikacin, and linezolid against *Nocardia* spp (*17*,*28*–*31*). However, in our study, *N. nova* complex was characterized by its susceptibility to clarithromycin and resistance to amoxicillin and clavulanate. This distinctive characteristic of *N. nova* complex is associated with the presence of membrane-bound penicillinase inducible by clavulinic acid (*17*,*27*). *N. veterana* had an antimicrobial drug susceptibility profile similar to that of *N. nova* complex. No minocycline resistance was observed, but 45% of isolates had intermediate susceptibility MICs, as has been reported (*32*–*34*).

CVC removal, along with the use of a combination of antimicrobial agents guided by antimicrobial drug susceptibility, should be the cornerstone of treatment of *Nocardia* bacteremia, particularly CLABSIs. We recommend using a combination of amikacin, carbapenems, and trimethoprim/sulfamethoxazole until the *Nocardia* isolate and its antimicrobial drug susceptibility can be determined (*17*,*32*,*33*).

In conclusion, *Nocardia* bacteria promote heavy biofilm formation in CVCs, and trimethoprim and minocycline in combination with anticoagulants as lock solutions have potent activity against *Nocardia* biofilm formation. In this study, *N. nova* complex isolates were the leading cause of *Nocardia* bacteremia. Isolation of *Nocardia* bacteria from the blood should always prompt consideration of *Nocardia* CLABSIs in cancer patients with indwelling CVCs, especially in the absence of signs and symptoms of pneumonia or disseminated infection.
